# Dextran sodium sulfate confounds causal role of periodontitis in inflammatory bowel disease

**DOI:** 10.1002/jper.70045

**Published:** 2025-12-09

**Authors:** Himanshi Tanwar, Jeba Mercy Gnanasekaran, Niyant Ganesh, Cindy Zhou, Amy Plotkin, Giacomo Baima, Massimo Costalonga, Fenfen Zhou, Hanping Feng, Priyam Jani, Jean‐Pierre Raufman, Vivek Thumbigere‐Math

**Affiliations:** ^1^ Division of Periodontology University of Maryland School of Dentistry Baltimore Maryland USA; ^2^ Department of Oncology and Diagnostic Sciences University of Maryland School of Dentistry Baltimore Maryland USA; ^3^ Pathology Biorepository Shared Service University of Maryland Greenebaum Comprehensive Cancer Center Baltimore Maryland USA; ^4^ Department of Surgical Sciences C.I.R. Dental School University of Turin Turin Italy; ^5^ Department of Diagnostic and Biological Sciences School of Dentistry University of Minnesota Minneapolis USA; ^6^ Department of Microbial Pathogenesis University of Maryland School of Dentistry Baltimore Maryland USA; ^7^ Department of Comprehensive Dentistry University of Maryland School of Dentistry Baltimore Maryland USA; ^8^ Division of Gastroenterology & Hepatology University of Maryland School of Medicine Baltimore Maryland USA; ^9^ Department of Biochemistry and Molecular Biology University of Maryland School of Medicine Baltimore Maryland USA; ^10^ Biomedical Laboratory Research and Development Service Veterans Affairs Maryland Healthcare System Baltimore Maryland USA; ^11^ Marlene and Stewart Greenebaum Comprehensive Cancer Center University of Maryland Medical Center Baltimore Maryland USA

**Keywords:** dextran sodium sulfate, DSS, inflammatory bowel disease, oral bacteria, oral‐gut axis, periodontitis, ulcerative colitis

## Abstract

**Background:**

Emerging evidence supports a bidirectional link between periodontitis and inflammatory bowel disease (IBD). To investigate this relationship, experimental models commonly use dextran sodium sulfate (DSS) to induce colitis. However, DSS is presumed to selectively affect the colon, and its potential off‐target effects on the oral cavity remain poorly understood. We examined whether DSS disrupts oral health, potentially confounding oral–gut axis research.

**Methods:**

C57BL/6 mice received 2% DSS in drinking water for 8 days, followed by 2 days of untreated water. Colitis severity was assessed by weight loss, colon length, histopathology, and quantitative real‐time–polymerase chain reaction (qRT‐PCR). Oral health was evaluated via micro–computed tomography (micro‐CT) analysis of alveolar bone, gingival histology, cytokine expression, and 16S rRNA sequencing of the oral microbiome.

**Results:**

DSS induced hallmark features of colitis, including weight loss, colon shortening, epithelial crypt damage, and mucosal inflammation. Strikingly, DSS also induced significant oral pathology, including alveolar bone loss, gingival epithelial hyperplasia, inflammatory infiltration, and upregulated gingival pro‐inflammatory cytokines (interleukin [IL] ‐6, IL‐17, tumor necrosis factor‐alpha [TNF‐α]). DSS further altered the oral microbiota causing reduced alpha‐diversity and a dysbiotic shift, with enrichment of *Streptococcus danieliae* and depletion of commensals such as *Lactobacillus murinus* and *Clostridium ASF502*. These microbial changes were accompanied by upregulated pathways involved in carbohydrate metabolism, oxidative stress response, and environmental sensing.

**Conclusion:**

DSS induces periodontal inflammation and oral dysbiosis, independent of colitis. These findings expose a critical confounder in oral–gut axis models and highlight the need to include DSS‐only or periodontitis‐only controls and alternative models to accurately distinguish systemic effects of DSS from true oral–gut interactions.

**Plain language summary:**

This study shows that dextran sodium sulfate (DSS), a chemical used to model gut inflammation in mice, also causes gum disease‐like changes—including bone loss, inflammation, and changes in oral bacteria. These findings reveal that DSS alone can affect the mouth and may confound studies investigating links between gum disease and inflammatory bowel disease, highlighting the need for better‐controlled models.

## 1 INTRODUCTION

As opposite ends of the orodigestive tract, the oral cavity and the intestine share anatomical, microbial, and immunological features that have bidirectional health implications. Compelling evidence suggests an association between periodontitis and inflammatory bowel disease (IBD).^1^, ^2^ IBD patients exhibit a higher incidence and severity of periodontitis, while individuals with chronic periodontitis are at increased risk of developing IBD.^2^, ^3^, ^4^, ^5^ Oral bacteria such as *Fusobacterium nucleatum*, *Porphyromonas gingivalis*, and *Prevotella intermedia* have been identified in the intestinal mucosa of IBD and colorectal cancer patients, where they have been implicated in epithelial barrier disruption, immune modulation, and in some cases, tumorigenesis.^6^, ^7^, ^8^, ^9^, ^10^ Since 2020, over 200 publications have reinforced the paradigm that the oral cavity is not merely a passive indicator of systemic health, but an active driver of gastrointestinal disease through microbial translocation, immune priming, and inflammatory amplification.^1^ Yet, the mechanistic underpinnings of the oral–gut axis remain poorly understood.

To explore these interactions, researchers have extensively relied on animal models. From more than 60 different experimental IBD models,^11^, ^12^, ^13^ the dextral sodium sulfate (DSS)‐induced colitis model has been widely used to investigate the potential causal link between periodontitis and IBD.^14^, ^15^, ^16^, ^17^, ^18^, ^19^, ^20^ DSS, a sulfated polysaccharide administered via drinking water, induces acute colonic injury by disrupting epithelial barrier integrity, thereby enabling translocation of luminal bacteria and antigens into the lamina propria and initiating an inflammatory response.^11^ This chemical model reliably recapitulates key features of human ulcerative colitis, such as bloody diarrhea, weight loss, and mucosal ulceration, with remarkable simplicity, rapidity, and reproducibility.^11^ By adjusting DSS concentration and duration, researchers can induce acute, chronic, or relapsing forms of colitis, making it a highly adaptable system for both pathogenesis studies and therapeutic testing.^11^ Many recent studies have combined DSS‐induced colitis with experimental periodontitis in mice to replicate the bidirectional disease state observed in humans.^14^, ^15^, ^16^, ^17^, ^18^, ^19^, ^20^ For example, mice with ligature‐induced periodontitis experience significantly worse colonic inflammation after DSS administration, echoing clinical observations that periodontitis can exacerbate IBD.^17^, ^19^, ^21^ Conversely, DSS‐induced colitis has been shown to aggravate periodontal disease in animal models, leading to increased gingival inflammation and alveolar bone loss, suggesting that intestinal inflammation can inversely drive oral pathology.^22^

However, the DSS model introduces a critical yet often‐overlooked confounder. Although DSS is administered to induce colonic inflammation, its potential off‐target effects on the oral cavity remain poorly understood. Prior studies investigating the periodontitis–IBD link have often lacked DSS‐only or periodontitis‐only controls, failing to establish whether DSS itself influences periodontal tissues, the oral microbiome, or oral immune cells.^14^, ^15^, ^16^, ^17^, ^18^, ^19^, ^20^ A recent systematic review and meta‐analysis highlighted this limitation, concluding that DSS can induce periodontitis‐like changes, rendering it “almost impossible” to distinguish DSS‐mediated pathology from true periodontitis‐driven colitis.^23^

To address this knowledge gap, we examined whether DSS alone is sufficient to induce pathological changes in the oral cavity. Using a standard 8‐day DSS regimen (2% wt/vol) largely employed in oral–gut axis studies,^14^, ^15^, ^16^, ^17^, ^18^, ^19^, ^20^ we found that DSS impacts oral health, leading to gingival inflammation and alveolar bone loss. Furthermore, DSS treatment shifted the oral microbiota toward a dysbiotic, pro‐inflammatory state. These findings demonstrate that DSS exerts direct effects on the oral cavity independent of intestinal inflammation, thereby posing a significant confounding variable in models studying oral–gut interactions. Our study highlights the need for DSS‐specific and periodontitis‐specific controls and consideration of alternative colitis models to ensure that the observed oral outcomes reflect true biological interactions rather than systemic artifacts of the experimental model.

## 2 MATERIALS AND METHODS

### 2.1 Animals

Eight‐week‐old wild‐type (WT) C57BL/6 mice (*n* = 6/group; 3 males, 3 females) were purchased from Inotiv (Lafayette, IN, USA) and acclimated for 2 weeks in a specific pathogen‐free (SPF) facility prior to experimentation. Mice were housed in sterile individually ventilated cages with ad libitum access to autoclaved chow and water. Animals were randomly assigned to DSS and control groups. Cage placement and order of sample collection were alternated to minimize environmental and handling confounders. Both male and female mice were included to account for sex as a biological variable. Mice were euthanized by CO_2_ inhalation.

The protocol was approved by the Institutional Animal Care and Use Committee (IACUC) of the University of Maryland Baltimore School of Medicine (Protocol Number: AUP#0420005) and conducted in accordance with the National Institutes of Health (NIH) Guide for the Care and Use of Laboratory Animals. This study adheres to the ARRIVE guidelines.

### 2.2 DSS–induced colitis model

**FIGURE 1:**
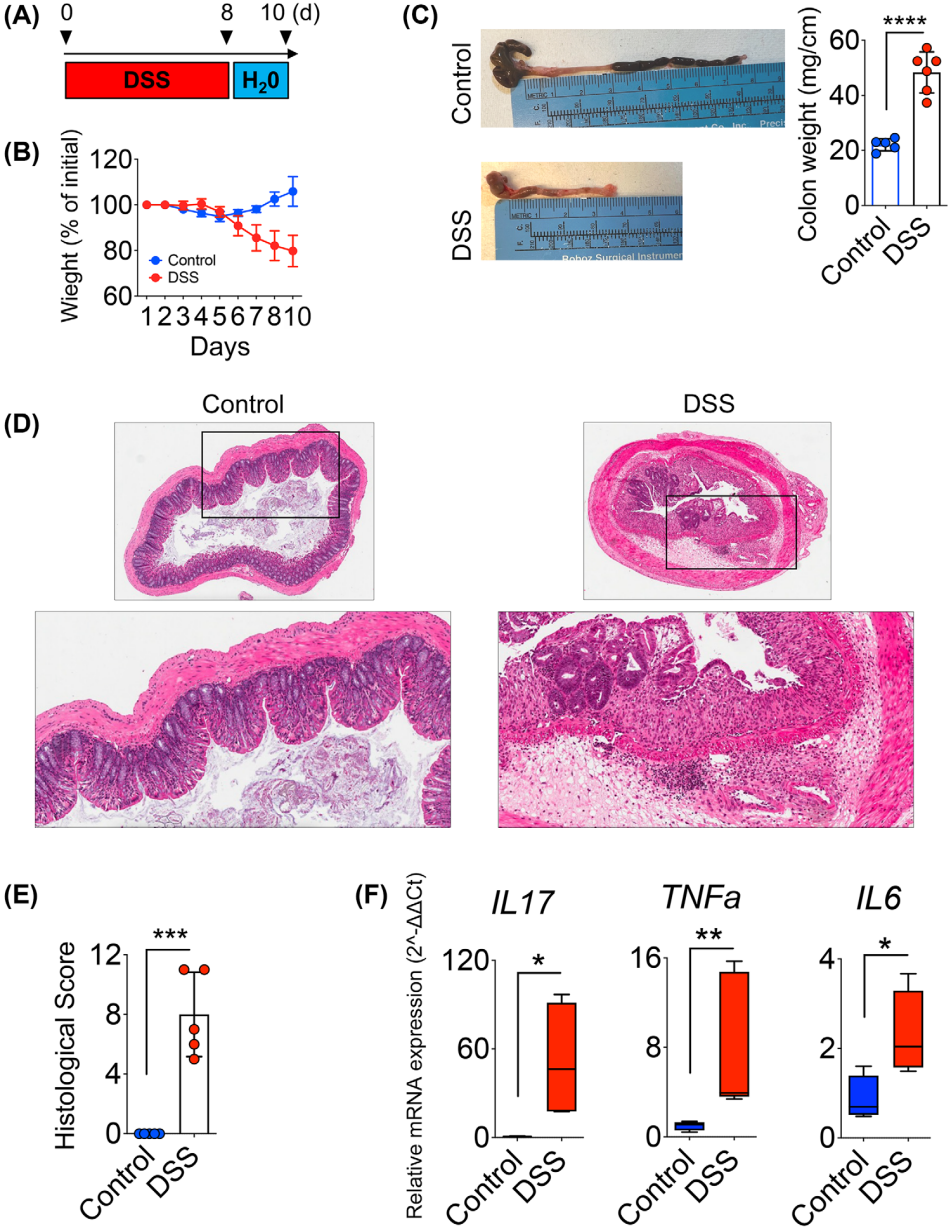
DSS Induces hallmark features of acute colitis in mice. (A) Experimental timeline showing administration of 2% DSS in drinking water for 8 days, followed by 2 days of regular water. Control mice received untreated drinking water throughout the experiment. (B) DSS‐treated mice exhibited progressive weight loss compared to weight gain in controls. (C) Representative images of colons harvested from control and DSS‐treated mice at necropsy. DSS colons were visibly shortened and thickened. (D) H&E‐stained cross‐sections of distal colon. DSS‐treated mice showed severe crypt loss, mucosal ulceration, goblet cell depletion, and dense inflammatory cell infiltration, in contrast to well‐preserved architecture in controls. (E) Histopathological scoring confirmed significantly elevated inflammation in DSS‐treated mice. (F) qRT‐PCR analysis of distal colonic mucosa showed upregulation of pro‐inflammatory cytokines (*n* = 5–6 mice/group; body weight analyzed by 2‐way repeated measures ANOVA; colon weight and cytokine expression compared using unpaired 2‐tailed *t*‐tests with Welch's correction or Mann–Whitney *U* tests depending on distribution; histological scores analyzed by Mann–Whitney *U* tests; data shown as mean ± SD or median [IQR] as appropriate; ***p < 0.001, **p < 0.01, *p < 0.05 and NS = non‐significant).

Colitis was induced by administering 2% (wt/vol) DSS (36,000‐50,000 M.Wt, MP Biomedicals, OH, USA) in drinking water ad libitum for 8 days, followed by 2‐days of untreated water^24^ (Figure 1A). Control mice received untreated drinking water throughout the experiment. All animals were provided fresh autoclaved food and water 3 times per week and monitored daily for body weight, food/water intake, stool consistency, and rectal bleeding. On day 10, animals were euthanized, and colons were excised, measured for length and weight, and processed for downstream analyses.^24^ Colon tissues were either fixed in formalin for histopathology or flash‐frozen for cytokine/chemokine analysis. Tongue samples were snap‐frozen in liquid nitrogen and stored at −80°C. Maxillae and mandibles were harvested and fixed in formalin for micro‐CT and histological analyses.

### 2.3 Ligature‐induced periodontitis model

A 5‐0 silk suture (Roboz) was tied around the maxillary left second molar for 10 days, with the contralateral unligated molar serving as an internal control.^25^ After 10 days, mice were euthanized, and gingival tissues harvested for quantitative real‐time–polymerase chain reaction (qRT‐PCR), and maxillary block biopsies collected for analysis of bone loss by micro–computed tomography (micro‐CT).

### 2.4 Micro‐CT analysis

Micro‐CT analysis was performed as previously described.^25^ Maxillae were scanned in a µCT 50 (Scanco Medical) at 70 kVp, 76 µA, 0.5 Al filter, with 900–1200 ms integration and 6–17 µm voxel dimension. DICOM files were created from raw data, exported, and calibrated to 5 known densities of hydroxyapatite (mg/cm^3^ HA). Reconstructed images were loaded and analyzed in AnalyzePro (version 14.0; AnalyzeDirect). Calibrated images were reoriented and analyzed by standard cortical and trabecular bone algorithms.^25^ Alveolar bone proper (ABP) was defined as bone within 240 µm of the tooth root, as measured radially from tooth root surfaces to include buccal, lingual, radicular, and interproximal alveolar bone surfaces. Alveolar bone loss was quantified around the first, second, and third maxillary molars on both right and left sides. For each animal, data from both sides were averaged and presented.

### 2.5 Histology

Mandibles were fixed overnight in Bouin's solution, demineralized in AFS (acetic acid, formaldehyde, sodium chloride) for 3–4 weeks, embedded in paraffin, and sectioned at 5‐µM thickness, as previously described.^25^ Hematoxylin and eosin (H&E) staining was performed on deparaffinized mandibular sections. Distal colonic tissues were fixed in Bouin's solution, paraffin‐embedded, sectioned at 5 µm, stained with hematoxylin and eosin (H&E), and examined under standard light microscopy. Histopathologic evaluation was conducted by a calibrated pathologist using a previously established scoring system based on 3 features^26^: Inflammation (0 = no significant inflammation; 1 = neutrophilic inflammation in epithelium or lamina propria; 2 = inflammatory cells extending into the submucosa; 3 = transmural inflammation); Crypt injury (0 = no crypt injury; 1 = loss of basal one‐third of crypts; 2 = loss of basal two‐thirds of crypts; 3 = loss of full thickness crypts; 4 = full thickness crypt loss with surface erosion; 5 = diffuse/confluent surface erosion), and Ulceration (0 = no ulceration; 1 = 2 or less foci of ulceration; 2 = 3 or 4 foci of ulceration; 3 = diffuse/confluent ulceration). Individual scores from each category were summed to yield a composite score ranging from 0 to 11 per mouse.^26^

### 2.6 qRT‐PCR

qRT‐PCR was performed as described previously.^25^ Briefly, total RNA was extracted using Trizol (Invitrogen), and cDNA was synthesized from 1 µg of total RNA using Superscript II RT (Invitrogen) with random hexamer primers (Promega). PCR was carried out using the QuantStudio‐3 Real‐Time PCR System (Applied Biosystems) according to the manufacturer's protocol. Transcript levels were normalized to the housekeeping gene *Hprt*, and the relative changes in gene expression were calculated using the 2^(‐delta delta C(t)) ^method.^25^ Primers sequences are listed in Table 1.

**FIGURE 2:**
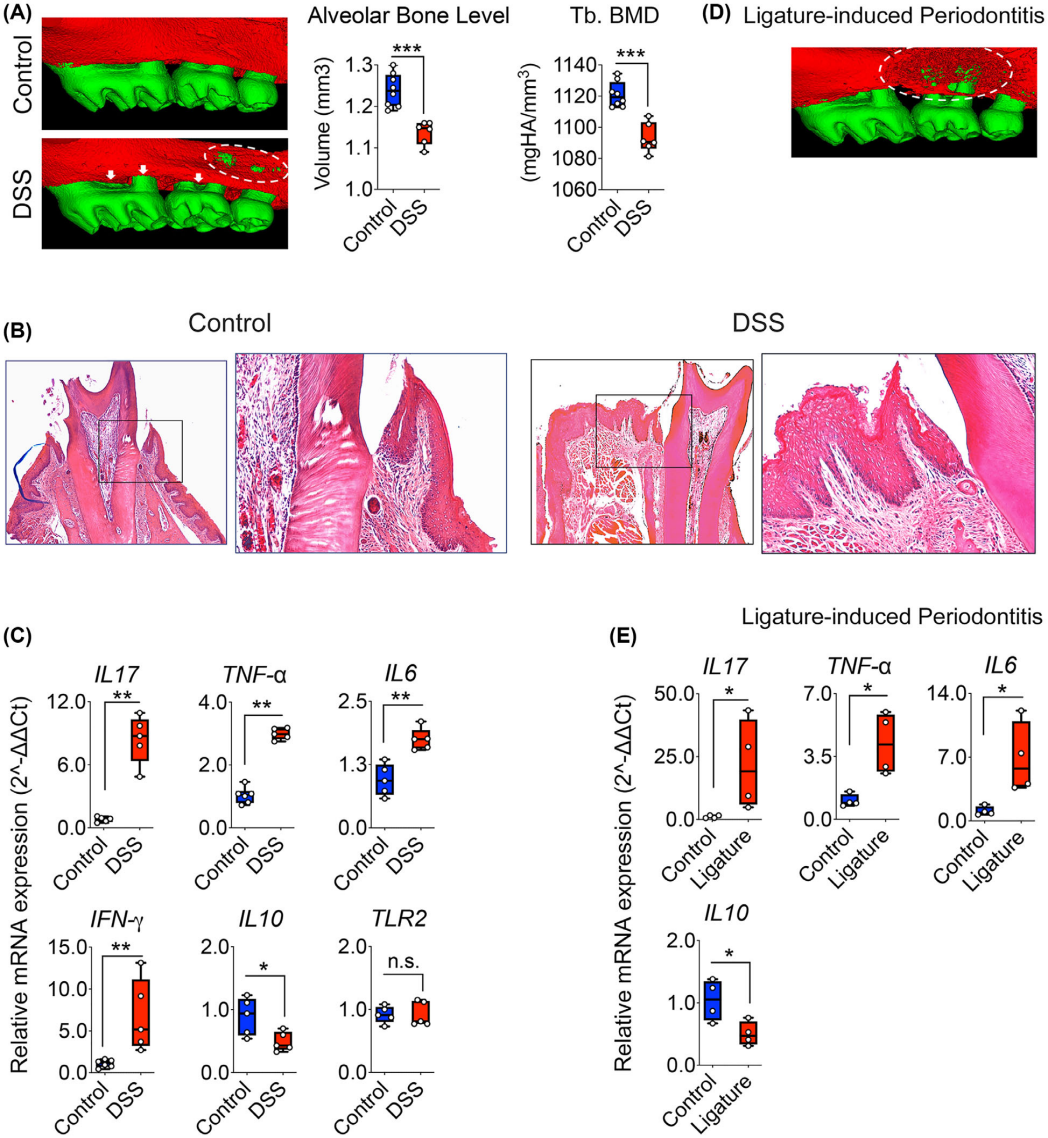
DSS induces periodontal inflammation and alveolar bone loss. (A) Representative micro‐CT reconstructions of maxillary alveolar bone from control and DSS‐treated mice. Quantification shows a significant reduction in alveolar bone volume (left) and trabecular bone mineral density (Tb. BMD, right) in DSS‐treated mice compared to controls. (B) H&E‐stained longitudinal sections of maxillary gingiva. Control tissues show a well‐organized, thin gingival epithelium and intact connective tissue architecture. In contrast, DSS‐treated tissues exhibit gingival epithelial thickening, irregular stratification, and elongation of rete ridges, consistent with epithelial hyperplasia. Subepithelial connective tissue is expanded with increased cellularity, indicative of inflammatory infiltrate. (C) The qRT‐PCR analysis of gingival tissue reveals upregulation of pro‐inflammatory cytokines and downregulation of anti‐inflammatory cytokine in DSS‐treated mice. (D) Representative micro‐CT image of ligature‐induced periodontitis showing focal vertical bone loss around the ligated maxillary second molar. (E) qRT‐PCR analysis of gingiva from ligated mice revealed stronger induction of pro‐inflammatory cytokines (*IL‐17, Tnfα, I‐L6*) and greater suppression of *IL‐10* compared to DSS, highlighting the greater severity of ligature‐induced periodontitis (*n* = 5–6 mice/group; data shown as mean ± SD or median [IQR] depending on distribution; significance determined by unpaired 2‐tailed *t*‐tests with Welch's correction or Mann–Whitney *U* tests as appropriate; ***p < 0.001, **p < 0.01, *p < 0.05, n.s. = not significant).

**TABLE 1:** qPCR primers related to Figures 1 and 2

Mouse *Hprt*	Forward	5′‐TCAGTCAACGGGGGACATAAA‐3′
	Reverse	5′‐GGGGCTGTACTGCTTAACCAG‐3′
Mouse *IL‐17A*	Forward	5′‐CAGACTACCTCAACCGTTCCAC‐3′
	Reverse	5′‐TCCAGCTTTCCCTCCGCATTGA‐3′
Mouse *TNFa*	Forward	5′‐GACCCTCACACTCAGATCATCTTCT‐3′
	Reverse	5′‐CCACTTGGTGGTTTGCTACGA‐3′
Mouse IL‐6	Forward	5′‐TCAGGAAATTTGCCTATTGAAAATTT‐3′
	Reverse	5′‐GCTTTGTCTTTCTTGTTATCTTTTAAGTTGT‐3′
Mouse INFG	Forward	5′‐GCTCTGAGACAATGAACGCT‐3′
	Reverse	5′‐AAAGAGATAATCTGGCTCTGC‐3′
Mouse IL‐10	Forward	5′‐ATTTGAATTCCCTGGGTGAGAAG‐3′
	Reverse	5′‐CACAGGGGAGAAATCGATGACA‐3′
Mouse TLR2	Forward	5′‐GTGCCACCATTTCCACGGGC‐3′
	Reverse	5′‐CAAAACACTTCCTGCTGGCC‐3′

### 2.7 16S RRNA SEQUENCING

Tongue tissues were aseptically collected, weighed, and homogenized. Genomic DNA was extracted using the DNeasy Blood & Tissue Kit (Qiagen). DNA concentration and purity were assessed using a NanoDrop spectrophotometer. The V1–V2 hypervariable region of the 16S rRNA gene was amplified using barcoded primers 27F (AGAGTTTGATCCTGGCTCAG) and 338R (TGCTGCCTCCCGTAGGAGT, and libraries were sequenced on an Illumina NovaSeq 6000 platform with PE250 reads (performed by Novogene). Raw sequencing reads were demultiplexed, quality‐filtered, denoised, and merged using the DADA2 pipeline.^27^ Amplicon sequence variant (ASV) reads were inferred and taxonomically assigned to species level using a 98% identity cutoff with mouse oral and NCBI 16S rRNA reference dataset.^28^, ^29^ Downstream analyses—including alpha and beta diversity metrics, differential abundance testing (e.g., ANCOM‐BC), and predictive functional profiling (e.g., via PICRUSt2)—were conducted on the resulting ASV table.^30^

### 2.8 Quantification and statistical analysis

Sample sizes were determined based on pilot data and power analyses. For the primary outcome measure of alveolar bone loss assessed by micro‐CT (BV/TV), pilot data indicated a 10% between‐group difference with a pooled standard deviation of 6%. Power analysis (2‐sided t‐test, *α *= 0.05, 80% power) estimated 5.6 mice/group; therefore, *n* = 6 mice/group (3 males, 3 females) were included. Secondary outcomes included colonic inflammation (weight loss, colon length, histopathology), gingival cytokine expression (qRT‐PCR), and oral microbiota composition (16S rRNA sequencing), which were analyzed for biological consistency but not individually powered.

Alveolar bone loss and cytokine levels were compared using unpaired 2‐tailed *t*‐tests. When equality of variances was rejected by *F*‐tests, Welch's correction was applied. Data distribution was assessed for normality using the Shapiro–Wilk test. When assumptions for normality and equal variance were met, results are reported as mean ± SD; when normality was not met, data are reported as median (interquartile range [IQR]) and analyzed using Mann–Whitney *U* tests. Data were analyzed using GraphPad Prism 9 (GraphPad Software, Inc.). Biological replicate numbers (*n*) are indicated in figure legends. Samples were excluded from analysis only in case of a clear technical problem that directly compromised endpoint measurements. Bone loss and histological analyses were performed in a blinded fashion, whereas the conduct of experiments and assessment of other outcomes were not blinded to the study personnel. Statistical significance was defined as *p* < 0.05.

## 3 RESULTS

### 3.1 DSS‐induced colonic inflammation

DSS treatment induced hallmark features of acute colitis in mice.^14^, ^15^, ^16^, ^17^, ^18^, ^19^, ^20^ Over the 8‐day course, DSS‐treated animals exhibited a progressive decline in body weight (−20%), in stark contrast to a slight weight gain in control animals (+6%) (*p* < 0.0001) (Figure 1B). Upon necropsy, we observed colon length was markedly shortened in DSS‐treated mice (mean 4.2 cm) compared to controls (mean 7.2 cm), representing a ∼42% reduction (*p* < 0.0001) (Figure 1C). Furthermore, DSS‐treated mice exhibited a ∼120% (*p* < 0.0001) increase in colon weight per unit length compared to controls (Figure 1C). The shortened, thickened, and hyperemic colons are consistent with epithelial injury and inflammatory remodeling, characteristic of DSS‐induced colitis.^14^, ^15^, ^16^, ^17^, ^18^, ^19^, ^20^

Histopathological examination of the distal colon revealed severe epithelial injury in DSS‐treated mice. Normal crypt architecture was nearly obliterated, with extensive mucosal ulceration, goblet cell depletion, and dense infiltration of neutrophils and mononuclear cells in the lamina propria and submucosa (Figure 1D). In contrast, control mice displayed intact epithelial crypts, preserved goblet cells, and minimal inflammatory cells. Quantitative histology scoring confirmed a significant increase in colitis severity in DSS‐treated mice (Figure 1E). qRT‐PCR analysis revealed upregulation of pro‐inflammatory cytokines in the colonic mucosa of DSS‐treated mice, including IL‐17 (∼42‐fold), tumor necrosis factor‐alpha (TNF‐α) (∼7‐fold), and interleukin (IL) ‐6 (∼2.0‐fold) (Figure 1F). Collectively, these findings establish that 2% DSS for 8 days induces robust colonic inflammation in WT C57BL/6 mice.

### 3.2 DSS promotes periodontal inflammation and alveolar bone loss

Remarkably, DSS also elicited profound effects on the oral cavity, despite no direct oral manipulation. Micro‐CT analysis of the maxillary arches revealed significant alveolar bone loss in DSS‐treated mice compared to controls, as evidenced by a ∼10% reduction in bone volume fraction (BV/TV; *p* = 0.004) and a ∼3% reduction in trabecular bone mineral density (Tb. BMD; *p* ≤ 0.001) (Figure 2A). Similar bone loss was observed in the mandible (data not shown), indicating a generalized skeletal response.

Histological examination of oral tissues from DSS‐treated mice revealed pronounced reactive epithelial changes and inflammation (Figure 2B). The gingival epithelium appeared thickened and irregularly stratified, with elongation of rete ridges—features consistent with acanthosis and epithelial hyperplasia. Subepithelial connective tissue appeared expanded with increased cellularity, suggestive of inflammatory infiltration. In contrast, control mice exhibited a thin, well‐organized epithelium with clear demarcation from the underlying connective tissue and no overt signs of inflammation.

qRT‐PCR analysis revealed upregulation of pro‐inflammatory cytokines in the gingiva of DSS‐treated mice, including IL‐17 (∼8.7‐fold), TNF‐α (∼3.0‐fold), IL‐6 (∼1.7‐fold), and interferon (IFN) ‐γ (∼6‐fold) (Figure 2C). In contrast, the anti‐inflammatory cytokine IL‐10 was downregulated (∼2.2‐fold) in DSS‐treated mice, while TLR2 expression remained unchanged. These cytokine changes in the periodontal tissue mirror the inflammatory milieu observed in the colon, suggesting that systemic immune activation and/or direct effects of DSS contribute to oral inflammation. While no overt ulcerations of the oral mucosa were observed, the histological and molecular evidence underscore significant subclinical inflammation in the periodontium. Collectively, these findings indicate that DSS alone is sufficient to induce periodontal inflammation and alveolar bone loss.

To contextualize DSS‐induced oral pathology, we compared it with the classic ligature‐induced periodontitis model (Figure 2D,E). Both models induced alveolar bone loss and gingival inflammation, but with distinct features. DSS caused diffuse moderate bone loss and moderate cytokine upregulation, whereas ligatures produced extensive focal vertical bone defects and robust inflammatory response. Thus, DSS recapitulates hallmark features of periodontitis in the absence of direct periodontal insult, underscoring its potential to confound oral–gut axis studies.

### 3.3 DSS promotes oral microbiome dysbiosis

**FIGURE 3:**
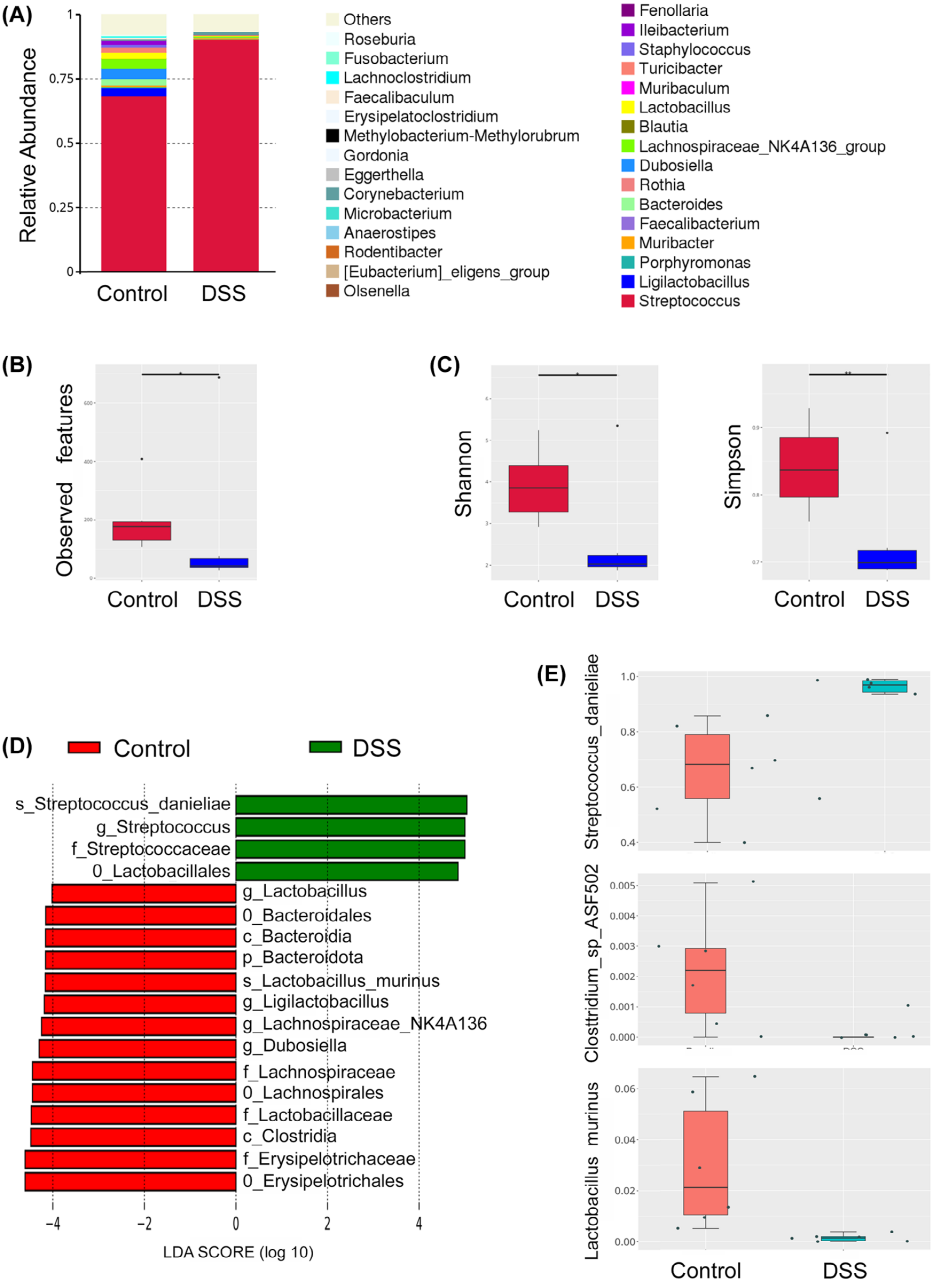
DSS induces oral dysbiosis and loss of microbial diversity. (A) Stacked bar plots of genus‐level taxonomic composition show major shifts in oral microbial communities between control and DSS‐treated mice. DSS treatment resulted in a marked expansion of *Streptococcus* species. (B,C) Alpha diversity metrics demonstrate a significant reduction in microbial richness and diversity in DSS‐treated mice. (B) Observed ASVs and (C) Shannon and Simpson diversity indices were significantly lower in DSS‐treated samples compared to controls. (D) LEfSe analysis identified differentially abundant taxa between groups. (E) Box plots of individual taxa confirm a significant increase in *Streptococcus danieliae* abundance in DSS‐treated mice, and depletion of *Clostridium sp. ASF502* and *Lactobacillus murinus* (*n* = 6 mice/group; alpha‐diversity analyzed by Wilcoxon rank‐sum; beta‐diversity assessed by PERMANOVA; differential taxa identified by LEfSe or ANCOM‐BC with multiple‐testing correction.

16S rRNA gene sequencing of oral samples revealed a profound shift in microbial community following DSS exposure, consistent with inflammation‐associated dysbiosis (Figure 3A). These changes parallel the loss of diversity often observed in the oral microbiota of IBD patients.^8^, ^9^ DSS treatment led to marked reduction in microbial diversity and richness. The number of observed ASVs was significantly lower in DSS samples compared to controls (Figure 3B), and both Shannon and Simpson indices confirmed a decline in alpha diversity (Figure 3C). Beta‐diversity analysis using principal coordinates analysis (PCoA) of Bray–Curtis dissimilarities showed a clear separation between DSS and control groups, indicating a shift in community composition (data not shown). This observation was statistically validated by PERMANOVA (Adonis), which showed that DSS treatment accounted for ∼20% of the variation in microbial composition (*R* ^2^ = 0.201), with a significant effect size (*F* = 2.52, *p* = 0.021).

**FIGURE 4:**
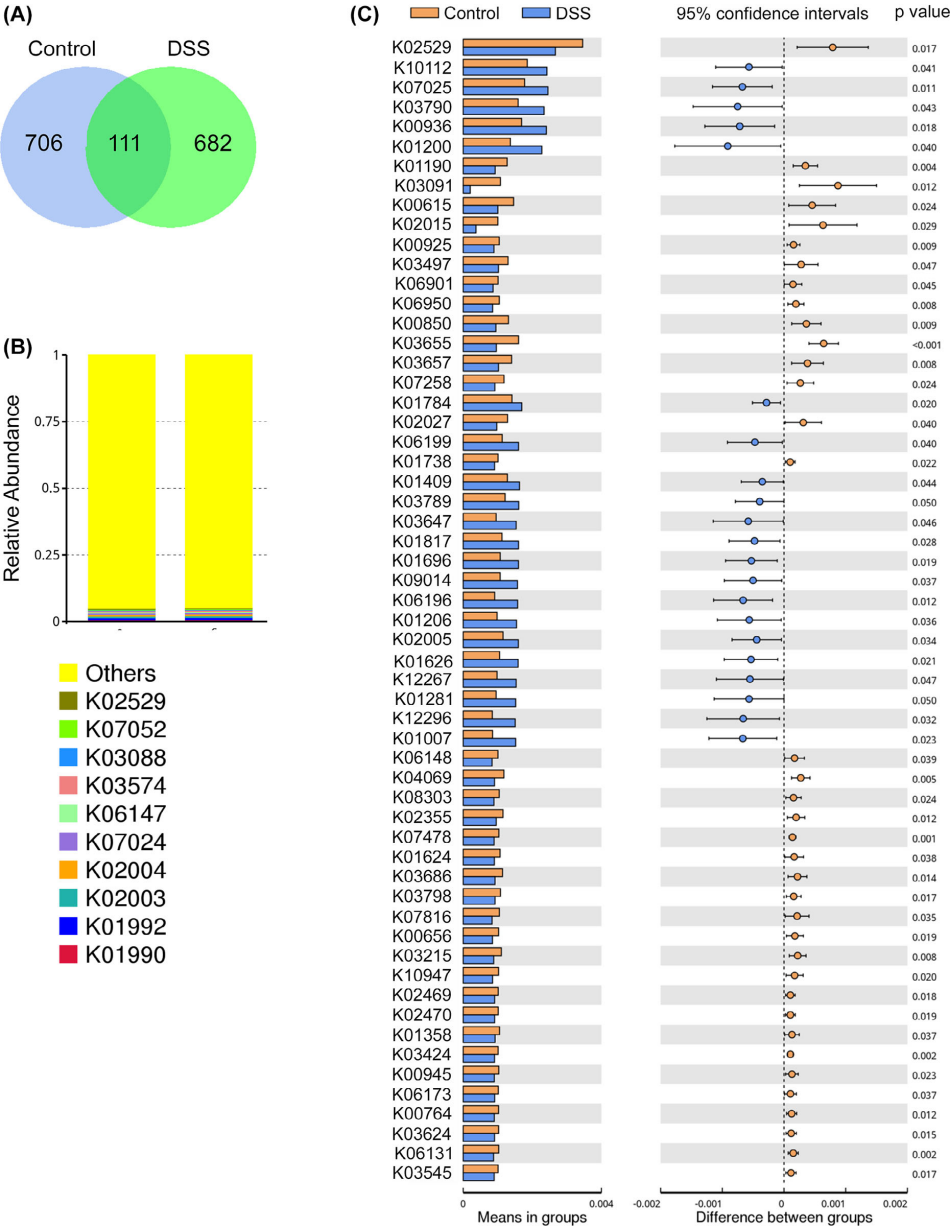
DSS treatment alters the functional potential of the oral microbiome. (A) Venn diagram illustrating amplicon sequence variants (ASVs) detected in control and DSS‐treated oral samples. (B) Stacked bar plot displaying the relative abundance of top enriched KEGG orthologs (KOs) in both groups based on PICRUSt2 functional prediction. (C) Differential abundance analysis of KOs between control and DSS groups. Bar plots (left) show mean abundance of KOs significantly altered between groups. Right panel shows 95% confidence intervals and adjusted *p*‐values for between‐group comparisons.

At the taxonomic level, DSS exposure caused a loss of beneficial commensals alongside an expansion of inflammation‐associated taxa. This ecological imbalance was characterized by a marked overrepresentation of *Streptococcus*, particularly *Streptococcus danieliae*, in DSS‐treated mice, while beneficial species such as *Lactobacillus murinus* and *Clostridium sp. ASF502* were markedly reduced (Figure 3D,E). The degree of microbial divergence was further evidenced by ASV comparison, where only 111 ASVs were shared between baseline and DSS groups, while 706 and 682 ASVs were unique to baseline and DSS, respectively—underscoring the extent of community restructuring (Figure 4A).

Functional predictions based on Kyoto Encyclopedia of Genes and Genomes (KEGG) orthology (using PICRUSt2) further revealed that DSS treatment significantly altered the functional potential of the oral microbiota (Figure 4B,C). Enriched pathways in DSS‐treated samples included those related to carbohydrate metabolism, such as starch and sucrose metabolism (K01200, pullulanase; K01206, α‐L‐fucosidase) and ABC sugar transporters (K10112). Notably, multiple enzymes involved in aromatic amino acid biosynthesis, including the tryptophan biosynthesis pathway (K01817, phosphoribosylanthranilate isomerase; K01696, tryptophan synthase β‐chain) and shikimate pathway (K01626, DAHP synthase), were also elevated.

DSS exposure further upregulated stress‐associated functions such as methionine sulfoxide reductase system (K12267, msrAB) and ribosomal protein acetylation (K03789, K03790), which are indicative of microbial adaptation to oxidative stress. Components of 2‐component signaling systems (K00936, PdtaS sensor kinase) and quorum sensing regulation (K12296, ComX peptide) were likewise enriched, suggesting enhanced environmental responsiveness in the dysbiotic community. Altogether, these functional shifts indicate that DSS reprograms the oral microbiota toward a stress‐adapted, metabolically versatile, and pro‐inflammatory state.

## 4 DISCUSSION

In this study, we demonstrate that DSS not only induces colonic inflammation but also triggers rapid and significant inflammatory changes in the oral cavity. As expected, DSS administration led to acute colonic inflammation characterized by weight loss, shortening of the colon, and the loss of colonic crypts. Strikingly, DSS also induced oral manifestations despite the absence of any direct oral insult. DSS‐treated mice exhibited significant alveolar bone loss and marked upregulation of inflammatory cytokines in gingival tissues, indicating that the oral mucosal immune milieu was activated systemically by DSS. Additionally, 16S rRNA sequencing revealed a dysbiotic shift in the oral microbiota, characterized by reduced alpha‐diversity, expansion of pro‐inflammatory taxa, and loss of commensals. Collectively, these findings establish that DSS exerts off‐target effects on the oral cavity.

To clarify the directionality of IBD‐to‐periodontitis outcomes, we found that DSS alone induces substantial periodontal changes, including alveolar bone loss, gingival epithelial hyperplasia, elevated pro‐inflammatory cytokines, and oral dysbiosis. These results confound the interpretation, since oral pathology can arise directly from DSS exposure independent of colitis activity. For periodontitis‐to‐IBD outcomes, DSS exerts a smaller but mechanistically relevant bias. DSS preconditions the oral environment through gingival inflammation and dysbiosis, reshaping the oral taxa available for gut translocation and colonization. Simultaneously, DSS disrupts colonic barrier integrity and primes mucosal immunity, which can both sensitize the colon to oral microbes and create ceiling effects on inflammation that blunt the apparent impact of periodontitis. Thus, while less pronounced than periodontitis‐to‐IBD outcomes, DSS still introduces systematic shifts that can inflate, mask, or misattribute the contribution of periodontitis to colitis severity.

Our results are consistent with previous observations and a recent meta‐analysis demonstrating that DSS damages both the intestine and the oral cavity, and can independently induce periodontitis‐like changes, making it difficult to distinguish DSS‐mediated pathology from true periodontitis‐driven colitis.^23^ Oz and Ebersole first reported that 2% DSS alone causes significant periodontal inflammation and alveolar bone loss in BALB/c mice, detectable by 7 weeks and progressing to severe periodontitis by 18 weeks.^31^, ^32^ This long‐term study concluded that DSS impacts mucosal tissue in a more generalized and systemic manner, affecting both the intestine and the oral cavity. Similarly, Mello‐Neto et al. reported that 3‐week DSS treatment in rats led to inflammatory infiltration of the periodontal connective tissues with marked upregulation of Th1/Th2‐related cytokines, including IL‐1α, IL‐1β, IL‐6, IL‐12, IL‐13, granulocyte–macrophage colony‐stimulating factor (GM‐CSF), IFN‐γ, and TNF‐α.^33^ They concluded that DSS should be used with caution as it can lead to more widespread and indiscriminate lesions, and the increased pro‐inflammatory cytokines in the gingival tissues caused by DSS might create an environment conducive to future alveolar bone loss. Consistent with these findings, we noted that such oral changes can arise as early as 1 week into DSS treatment, underscoring the rapid systemic impact of DSS. Additionally, Metzger et al.^34^ and Hamdani et al.^35^ demonstrated that 2‐weeks of DSS treatment suppresses bone formation and increases bone resorption, resulting in reduced bone mass and altered bone microarchitecture. In DSS‐treated mice, elevated TNF‐α, IL‐6, RANKL, OPG, and sclerostin corresponded with higher osteoclast surfaces and lower rates of bone formation.^34^, ^35^

Beyond immune and bone phenotypes, DSS also perturbs the composition and functional capacity of the oral microbiota. Rautava et al. showed that even a brief, 1‐week course of 2% DSS altered the oral microbiome composition of C57BL/6 mice, particularly reducing health‐associated taxa such as Spirochaetes, Betaproteobacteria, and Lactobacillus.^36^ These shifts were more evident in saliva than in tongue or buccal mucosa, and it occurred in the absence of visible oral inflammation.^36^ Similarly, we observed oral dysbiosis within 1 week of DSS treatment, marked by reduced bacterial diversity, depletion of beneficial taxa such as *L. murinus* and *Clostridium* sp. ASF502, and enrichment of *S. danieliae*, a core murine oral commensal.^28^ Although not inherently pathogenic, *S. danieliae* can act as a context‐dependent pathobiont. Its oral administration after antibiotic pretreatment exacerbates imiquimod‐induced psoriasis‐like dermatitis in mice with increased TNF‐α, IL‐17, and IL‐22,^37^ and its enrichment in IgA nephropathy models correlates with epithelial barrier dysfunction.^38^ Functional metagenomic predictions in our study revealed enrichment of carbohydrate‐metabolism and oxidative‐stress pathways (e.g., pullulanase, α‐L‐fucosidase, msrAB) in DSS‐treated mice, suggesting enhanced metabolic adaptability and stress tolerance typical of commensal‐to‐pathobiont transitions. In the DSS‐primed host, marked by epithelial barrier compromise and systemic cytokine activation, the altered ecological niche likely favors facultative pathobionts capable of amplifying mucosal inflammation, thereby lowering the threshold for periodontal tissue breakdown. Notably, these dysbiotic changes occurred in the absence of any exogenous microbial challenge, confirming that DSS alone, via its systemic inflammatory effects, is sufficient to perturb the oral microbial ecosystem and potentiate inflammation‐driven damage.

While prior studies have reported that prolonged DSS exposure (≥3 weeks) can affect the oral cavity, these models were primarily designed to assess systemic outcomes or late‐stage colitis. No study has simultaneously assessed bone loss, gingival inflammation, cytokine expression, and microbiome dysbiosis within a single animal model—particularly in C57BL/6 mice, the predominant strain used in periodontitis–IBD studies. By integrating these endpoints in a standard, short‐term regimen widely used in oral–gut axis research, our study offers the first comprehensive, mechanistic evaluation of DSS's off‐target effects on the oral cavity. This within‐cohort approach reduces inter‐animal variability and yields biologically meaningful insights into host–microbiome interactions. Given the recent surge in oral–gut axis research, our study provides much‐needed mechanistic clarity and experimental rigor, highlighting the need to re‐evaluate widely accepted models that may mistakenly attribute DSS effects to true oral–gut interactions.

Importantly, these findings raise critical concerns about the interpretive validity of DSS‐based models in oral‐gut axis studies. DSS is the most widely used drug to induce ulcerative colitis in rodents and is frequently employed in studies exploring the bidirectional relationship between periodontitis and IBD.^14^, ^15^, ^16^, ^17^, ^18^, ^19^, ^20^ However, our data clearly demonstrate that DSS alone can trigger periodontal inflammation and bone loss, which means that studies lacking appropriate controls could risk misattributing DSS's direct effects on the oral cavity to an interplay between periodontitis and IBD. Many previous studies examining oral–gut connections have often assumed that the observed oral changes stem solely from induced periodontitis, without accounting for the intrinsic oral effects of DSS.^14^, ^15^, ^16^, ^17^, ^18^, ^19^, ^20^ For instance, “2‐hit” models combining ligature‐induced periodontitis with DSS‐induced colitis have been interpreted as evidence of synergistic worsening of colitis by periodontitis‐related oral bacteria.^17^, ^19^, ^21^ However, based on our findings and those of others, it is conceivable that some of the oral pathology and even part of the exacerbation of colitis in those models were due to DSS's intrinsic ability to alter oral immunity and oral microbiota.

DSS‐induced oral mucosal inflammation may create a conducive environment for the expansion of opportunistic pathogens that are not typically central to periodontitis pathogenesis. For example, in DSS‐treated mice subjected to oral ligatures, *Klebsiella aerogenes* and *Klebsiella pneumoniae* have been found to overgrow in the oral cavity and subsequently translocate to the gut, exacerbating colonic inflammation.^17^ However, these species are generally considered as opportunistic pathogens and are typically found at low abundance (1.9% and 1.5‐8.5%, respectively) in the subgingival biofilm of periodontitis patients.^39^, ^40^, ^41^, ^42^ They are not recognized as primary drivers of periodontitis. Indeed, their abundance remains low in mice subjected to ligature‐induced periodontitis alone, suggesting that DSS‐induced oral inflammation may facilitate their selective expansion.^17^ This raises the possibility that DSS may confound the oral environment, enabling the proliferation of noncanonical species that are then mistakenly interpreted as key drivers of periodontitis and its systemic consequences on the intestine Notably, Klebsiella species are not found at high abundance in the oral cavities of IBD patients with periodontitis,^43^, ^44^, ^45^ further emphasizing the risk of misinterpretation when DSS‐based models are used without adequate controls. Moreover, a careful examination of previous studies reveals that while DSS significantly induces colitis, the subsequent colitis exacerbation by oral bacteria is comparatively minimal,^16^, ^20^ which cautions against overinterpretation of causality in periodontitis–IBD studies utilizing DSS.

Despite these limitations, the DSS model remains valuable for identifying potential links, but it should be complemented by alternative approaches for a more comprehensive understanding. Future studies on periodontitis–IBD interactions must account for DSS as a potential confounder. Without appropriate DSS‐only and periodontitis‐only control groups, one cannot discern whether the observed oral pathology is truly due to oral–gut crosstalk or simply a direct consequence of DSS exposure. We therefore interpret our findings as a cautionary note: conclusions from prior DSS‐based oral–gut studies may benefit from cautious reinterpretation, with greater consideration of DSS's intrinsic effects to minimize misattribution of causality. Our findings highlight the need for refined experimental models and study designs to dissect the bidirectional oral–gut relationship with greater fidelity to human disease. Below we outline recommendations for future research:

- **Include DSS‐Only and Periodontitis‐Only Control Groups**: Any study using DSS to investigate periodontitis–IBD interactions should include DSS alone (without induced periodontitis) and periodontitis alone (without DSS) control groups. These baseline groups are essential to differentiate oral and gut changes directly caused by DSS from those arising through oral–gut interplay.
- **Use Alternative Chronic IBD Models**: DSS is never prescribed to humans. To better mimic the chronic, immune‐mediated nature of human IBD and its oral manifestations, researchers should consider chemicals that are commonly prescribed to humans or use non‐chemical colitis models. For example, non‐steroidal anti‐inflammatory drugs (NSAIDs) increase the risk of IBD by affecting intestinal mucosal barrier function, mainly through inhibition of cyclooxygenase enzymes and colonic levels of prostaglandin E2.^46^, ^47^ NSAIDs like piroxicam may offer more translatable insights into IBD pathogenesis,^48^, ^49^ provided they do not have any off‐target effects on the oral cavity. Genetic models such as IL‐10 knockout mice (exhibiting chronic, Th1/Th17‐driven colitis) and the T cell transfer model (adaptive immunity‐mediated colitis) might more closely mimic human disease.^17^, ^50^ Spontaneous ileitis models like SAMP1/Yit^Fc mice, which also exhibit periodontal bone loss, further support their utility in oral–gut axis studies.^51^
- **Utilize Microbiota‐Humanized and Gnotobiotic Approaches**: Given the microbiome's central role, future studies should employ germ‐free or microbiota‐humanized mice to dissect microbial contributions. Colonizing germ‐free mice with oral bacteria from periodontitis patients or gut microbiota from IBD patients can recreate a more human‐relevant oral–gut axis. This also enables controlled testing for how specific microbial communities (oral, gut, or both) drive inflammation in the opposite compartment. It also allows to investigate if interventions (e.g., probiotics, fecal transplants) can restore microbial balance and ameliorate inflammation.
- **Incorporate Integrated Multi‐Organ Monitoring**: Studies should longitudinally monitor oral and gut parameters (cytokines, histology, microbial profiles) in parallel. Omics‐based time‐course analyses could identify predictive biomarkers—for example, gingival cytokine spikes preceding gut flares—clarifying the sequence and directionality of events. This systems‐level approach would help map causal links and inform targeted interventions.

## 5 CONCLUSIONS

In conclusion, our study has several important implications. First, it encourages the need for cautious, context‐specific reinterpretation of DSS‐based animal studies on periodontitis–IBD interactions, particularly in cases where oral endpoints were limited or control groups were incomplete. It is possible that some conclusions (e.g., the extent to which periodontitis affected colitis outcomes) were confounded by unrecognized DSS‐induced oral pathology. Moving forward, future studies must incorporate appropriate controls and consider complementary models to help delineate DSS‐specific effects from true oral–gut cross‐talk. Such refinements will strengthen the reproducibility, translational relevance, and mechanistic clarity of research into the bidirectional link between periodontitis and IBD.

## AUTHOR CONTRIBUTIONS

V.T.M., H.T., and J.M.G. conceived the project and designed the experiments; H.T., J.M.G., and F.F.Z. performed DSS‐induced colitis studies; N.G. and P.J. performed micro‐CT analyses; V.T.M. performed microbial analyses; A.P. performed histological analysis; C.Z. performed histology sectioning; G.B., M.C., H.F., and J.P.R. advised on experiments and provided intellectual input; V.T.M. wrote the paper and supervised the project; all authors contributed to reviewing and editing.

## CONFLICT OF INTEREST STATEMENT

Dr. Massimo Costalonga is an Associate Editor for the Journal of Periodontology and a co‐author of this article. To minimize bias, all authors were excluded from all editorial decision‐making related to the acceptance of this article for publication.

## Data Availability

The accession number for the 16S rRNA sequencing dataset reported in this paper can be found at BioProject accession: PRJNA1289225 (https://dataview.ncbi.nlm.nih.gov/object/PRJNA1289225?reviewer=j007e1el96obpd656pcbv58s89).
